# Analyzing global research trends and focal points of pyoderma gangrenosum from 1930 to 2023: visualization and bibliometric analysis

**DOI:** 10.1186/s12967-024-05306-4

**Published:** 2024-05-27

**Authors:** Sa’ed H. Zyoud

**Affiliations:** 1 Department of Clinical and Community Pharmacy, College of Medicine and Health Sciences, An-Najah National University, Nablus, 44839 Palestine; 2 Clinical Research Centre, An-Najah National University Hospital, Nablus, 44839 Palestine

To the Editor, I read with great interest the publication entitled “An approach to the diagnosis and management of patients with pyoderma gangrenosum from an international perspective: results from an expert forum” [1]. Pyoderma gangrenosum is an ulcerative, cutaneous condition with distinctive clinical characteristics first described in 1930 [2]. Due to the importance of the subject, this published study was searched in databases, and I did not find any bibliometric studies on this topic. In recent years, researchers have successfully applied bibliometric analysis in various domains, contributing to the development of novel theories and assessing research frontiers, including in the dermatology field. Nonetheless, comprehensive bibliometric analyses of P. gangrenosum have not been performed. This study addresses this gap by conducting a thorough bibliometric analysis in the field of P. gangrenosum at the global level. The goal is to assist researchers in swiftly grasping the knowledge structure and current focal points in the field, generating new research topic ideas, and enhancing the overall quality of research on P. gangrenosum.

This bibliometric analysis sought to delineate research endeavors concerning P. gangrenosum, pinpoint the primary contributing countries, and discern prevalent topics within this domain. Using a descriptive cross-sectional bibliometric methodology, this study extracted pertinent documents from the Scopus database covering the period from 1930 to December 31, 2023. The search strategy included keywords related to ‘pyoderma gangrenosum.’ VOSviewer software (version 1.6.20) was used to illustrate the most recurring terms or themes [3]. The scope of the retrieved documents was restricted to including only journal research articles while ignoring other forms of documents.

Overall, 4,326 papers about P. gangrenosum were published between 1930 and 2023. Among these were 3,095 (71.54%) original papers, 548 (12.67%) letters, 477 (11.03%) reviews, and 206 (4.76%) other kinds of articles, such as conference abstracts, editorials, or notes. With 3,454 publications, English was the most often used language, followed by French (*n* = 253), German (*n* = 190), and Spanish (*n* = 163), accounting for 93.85% of all related publications.

Figure 1 shows the distribution of these publications. Between 1930 and 2023, there were steadily more publications on P. gangrenosum (R^2^ = 0.9257; P value < 0.001). Growth trends and productivity trends in P. gangrenosum-related publications have been influenced by developments in medical research, clinical practice and patient care [4, 5]. All of these factors have advanced our knowledge of the condition, enhanced our methods of treatment, and helped to create standardized findings for clinical studies.

**Fig. 1:**
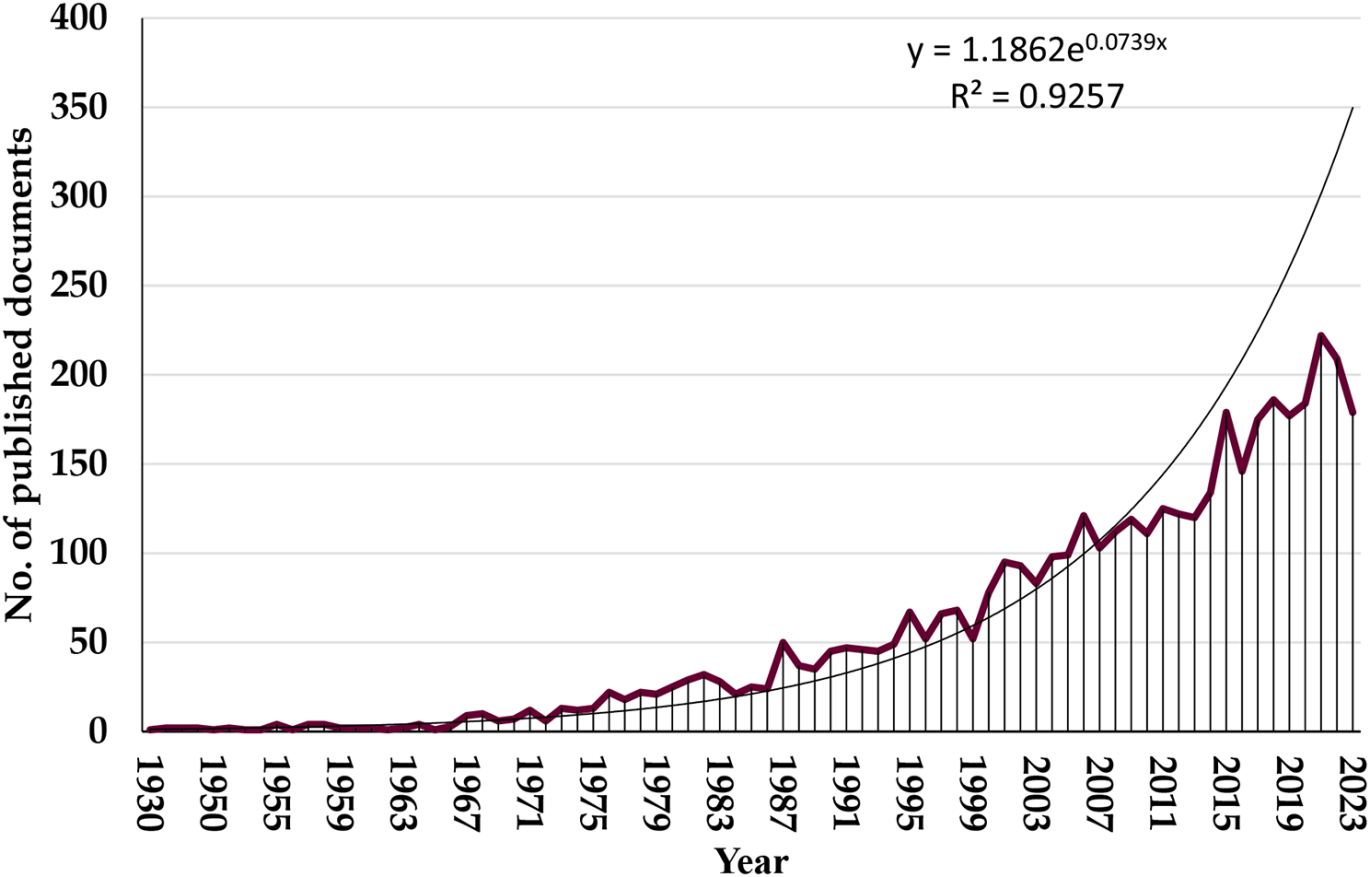
Annual growth of published research related to P. gangrenosum (1930–2023)

The top 10 countries with the most publications on P. gangrenosum are listed in Table 1. These are the USA (*n* = 1073; 24.80%), the UK (*n* = 345; 7.98%), Japan (*n* = 335, 7.74%), and Germany (*n* = 296; 6.84%). With 65 articles, the Mayo Clinic in the USA led the institutions; Oregon Health & Science University in the USA and Università degli Studi di Milano in Italy followed with 60 articles each.

**Table 1:** Ten leading countries in publications concerning pyoderma gangrenosum

Ranking	Country	No. of documents	%
1st	United States	1073	24.80
2nd	United Kingdom	345	7.98
3rd	Japan	335	7.74
4th	Germany	296	6.84
5th	Italy	280	6.47
6th	France	267	6.17
7th	Spain	187	4.32
8th	Turkey	122	2.82
9th	India	117	2.70
10th	Canada	106	2.45

To create a term co-occurrence map in VOSviewer 1.6.20, terms had to appear in the title and abstract at least forty times by binary counting. The network was visualized by building the map using terms with the highest relevance scores. Large bubbles for often cooccurring terms and close spacing between terms with high similarity were guaranteed by the algorithm. The larger circles in Fig. 2A represent frequently occurring terms in titles and abstracts. Four primary topic clusters—“Treatment modalities” (green cluster), “epidemiology and clinical presentation” (blue cluster), “improved diagnostic methods” (red cluster), and “the links between P. gangrenosum and other morbidities such as inflammatory bowel disease or autoimmune conditions” (yellow cluster)—are distinguished by color.

**Fig. 2:**
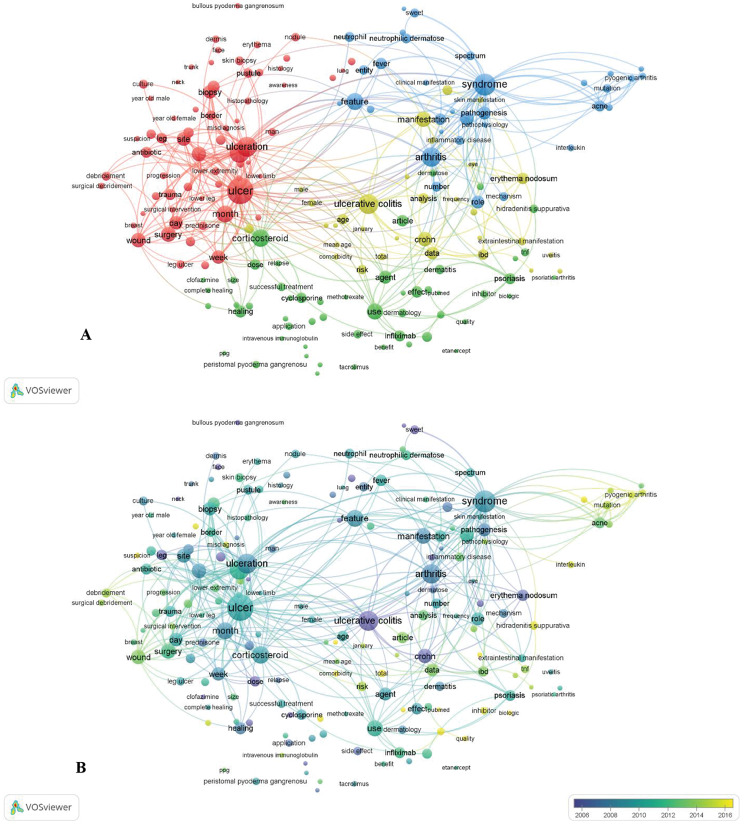
Mapping of terms used in research on P. gangrenosum. **A**: The co-occurrence network of terms extracted from the title or abstract of at least 40 articles. The colors represent groups of terms that are relatively strongly linked to each other. The size of a term signifies the number of publications related to P. gangrenosum in which the term appeared, and the distance between two terms represents an estimated indication of the relatedness of these terms. **B**: Mapping of terms used in research on P. gangrenosum. The terms “early” (blue) or “late” (red) years indicate when the term appeared

Interestingly, after 2012, terms related to “treatment modalities” and “epidemiology and clinical presentation” have gained more attention than in the past, which focused on “improved diagnostic methods” and “the links between P. gangrenosum and other morbidities such as inflammatory bowel disease or autoimmune conditions” (pre-2012). Figure 2B shows this tendency.

In conclusion, there has recently been an increase in P. gangrenosum research, especially in the last decade. The current focus of research is on treatment challenges, obstacles to diagnosis, and connections to underlying diseases. Furthermore, efforts are being made to create core outcome sets and standardized diagnostic criteria for clinical trials. These patterns demonstrate continuous attempts to comprehend, identify, and treat this illness with greater effectiveness. This recent increase in research has important implications for clinical practice. Clinicians can improve patient care by remaining current in emerging trends and areas of interest. Moreover, an in-depth analysis of previous studies can identify knowledge gaps, directing future research efforts toward the most important issues. In the end, a deeper comprehension of the body of research can result in better clinical judgment based on best practices, which could enhance patient outcomes and advance the dermatological field.

## Data Availability

This published article contains all the information produced or examined in this research. Additional datasets utilized during this study can be obtained from the corresponding author.
